# Dietary Fe-Gly supplementation attenuates enterotoxigenic *Escherichia coli* (ETEC)-induced inflammation response and intestinal barrier dysfunction in piglets

**DOI:** 10.3389/fvets.2025.1537604

**Published:** 2025-01-29

**Authors:** Qing Gao, Yilong Zhang, Yabin Wu, Dianchao Gu, Junzhou Chen, Conghui Yin, Hao Wu, Dan Zhu, Daiwen Chen, Aimin Wu

**Affiliations:** ^1^College of Animal Science, Xichang University, Xichang, Sichuan, China; ^2^Animal Nutrition Institute, Key Laboratory for Animal Disease-Resistance Nutrition, Ministry of Education, Ministry of Agriculture and Rural Affairs, Key Laboratory of Sichuan Province, Sichuan Agricultural University, Chengdu, China; ^3^Hunan Debon Biotechnology Co., Ltd., Changning, Hunan, China; ^4^Tongwei Agricultural Development Co., Ltd., Chengdu, Sichuan, China

**Keywords:** iron, piglets, enterotoxigenic *Escherichia coli* (ETEC), inflammation response, intestinal barrier

## Abstract

Iron in the animal gut that is not utilized by the host can be directly utilized by microorganisms, particularly harmful ones. Organic iron (such as Fe-Gly) has high digestive and absorption efficiency in the body. It is currently unclear whether it can reduce the utilization of iron by ETEC, thereby mitigating the harm caused by ETEC infections. This experiment mainly studies the effects of adding Fe-Gly to the diet on the growth performance, iron nutritional status, and intestinal morphology of weaned piglets infected with ETEC. The study found that adding 50 mg of Fe-Gly to the diet significantly increased ADFI and ADG by 30.6 and 35.3%, respectively (*p* < 0.05), and alleviated the issues of diarrhea and reduced growth performance caused by ETEC infection. The diarrhea rate decreased by 40% (from 31.25 to 18.75%). In addition to protecting the health of piglets, adding Fe-Gly can also increase the TIBC level in piglet serum (*p* < 0.05), enhancing their ability to bind and transport iron. From the gene expression results and tissue section results, adding Fe-Gly can also alleviate the damage to the jejunum caused by ETEC challenge to some extent (*p* < 0.05). In conclusion, adding 50 mg of Fe-Gly can meet the daily needs of piglets, improve iron utilization efficiency, and reduce the residual iron in the intestines. This decreases the iron available for pathogenic microorganisms in the gut, thereby inhibiting the proliferation of intestinal pathogens and ensuring the intestinal health of piglets.

## 1 Introduction

Iron is an important nutrient for piglets, iron deficiency seriously affects the normal growth of piglets, resulting in loss of appetite, slow growth, and many other undesirable symptoms. Additionally, iron deficiency can reduce the piglet’s ability to resist disease, reduce the piglet’s disease resistance by decreasing specific immune cell numbers, thereby compromising cellular immunity, and affecting the humoral and non-specific immunity (1). Only 5–20% of the iron ingested through feed is absorbed by the duodenum. About 80% of the ingested iron is retained in the intestinal lumen for microbial absorption and use (2). This is because iron is a protein cofactor involved in key metabolic pathways, and the replication and growth of almost all bacteria in the intestinal microbiota depend on this unabsorbed iron in the intestinal lumen (3, 4). Thus, it is clear that iron is closely related to microbial proliferation, and increasing the efficiency of the body’s absorption of ingested iron, reducing the unabsorbed iron in the intestinal lumen may be an effective antimicrobial strategy to benefit piglet health.

Currently, ferrous sulfate (FeSO_4_) is the most commonly used form of iron in feed. However, inorganic iron has several disadvantages, including chemically unstable, less absorption efficiently, and a tendency to accelerate the oxidation and rancidity of lipids in the feed. Ding et al. found that excessive addition of FeSO_4_ in the diet significantly reduced the villus height of the duodenum, jejunum, and ileum in weaned piglets, damaging the intestinal morphology. Furthermore, excessive FeSO_4_ significantly inhibited the expression of tight junction proteins in the duodenum, increased intestinal permeability, and caused mitochondrial swelling in the duodenum, thereby impairing the repair and regeneration capacity of the intestinal mucosa (5). Therefore, when using inorganic iron as an iron source supplement for piglets, the amount added tends to be much higher than the actual requirement, which not only raises the cost of feed production, but also increases the source of iron available to microorganisms in the gut and raises the risk of intestinal disease. In addition, the iron that is eliminated from the body with feces can also pollute the environment. In recent years, many studies have found that amino acid chelated iron is structurally stable, does not form ionic iron, and reduces the effects of various anti-nutrients (6). Iron supplemented in the form of amino acid chelated iron can be directly absorbed by the body and transported to specific sites to perform its functions, reducing intermediate processes and thus improving bioavailability efficiency. Some studies have shown that the absorption rate of amino acid chelated iron is 2–4 times higher than that of FeSO_4_, and that Fe-Gly enters the cellular mucosa in a chelated form (7–10). Ma et al. found that in the Caco-2 cell model, the Fe-Gly translocation rate across the cell monolayer was found to be significantly higher than that of FeSO_4_ (11). Iron glycinate (Fe-Gly), as a kind of amino acid chelated iron, has the advantages of amino acid chelated iron, and is a kind of efficient iron supplement additive (12). Therefore, we hypothesize that the addition of FeSO_4_ and Fe-Gly at equivalent doses may significantly increase iron absorption and reduce iron levels in the cecum and colon, thereby inhibiting the proliferation of harmful microorganisms. This experiment uses FeSO_4_ as a control and aims to investigate the effects of low-dose Fe-Gly addition on the production performance of weaned piglets, as well as its intervention effects on the intestinal damage and disorders caused by ETEC infection.

## 2 Materials and methods

The animal experiment in this study was carried out after approved by the Animal Care and Use Committee of Sichuan Agricultural University (Chengdu, China, No. 20210111).

### 2.1 Experimental materials

Fe-Gly, FeSO_4_, and other trace elements were purchased from Hunan Depon Biotechnology Co. Feed ingredients: corn, wheat bran, fish meal, rock flour, calcium bicarbonate, sodium chloride, synthetic amino acids, choline chloride (from Sichuan Agricultural University Teaching and Research Base); peeled soybeans, expanded soybeans, soybean oil, sucrose, glucose, enzyme preparation (purchased from Sichuan Chiyang Agricultural and Animal Husbandry Co., Ltd).

### 2.2 Animal experiments

In this experiment, 32 weaned piglets with an average body weight of 7.08 kg at 21 days of age were selected. After 3 days of pre-feeding, they were weighted and divided into 4 treatment groups using randomized block group design, with 8 replicates each, 1 piglet in each replicate (Table 1). The piglets were fed diets containing 100 mg/kg of FeSO_4_ and 50 mg/kg of Fe-Gly, respectively. The amount of FeSO_4_ added is based on the standards set by the NRC (2012), while the Fe-Gly reference is derived from previous studies (9). The test period lasted 21 days, during which the piglets were fed and watered libitum. At the end of the test, the piglets were challenged with ETEC by oral gavage, while the control group received the same volume of LB medium. The piglets were observed for diarrhea after the challenge, and sampling was performed when diarrhea was evident in the two treated groups.

**Table 1:** Experimental design.

Treatment	Diets	Number of piglets
T1	FeSO_4_ (100 mg Fe/Kg)	8
T2	Fe-Gly (50 mg Fe/Kg)	8
T3	FeSO_4_ (100 mg Fe/Kg) + ETEC	8
T4	Fe-Gly (50 mg Fe/Kg) + ETEC	8

### 2.3 Piglet test diets

The diets in this trial were configured with reference to the NRC (2012) nutritional requirements for piglets in the 5–11 kg stage (Table 2).

**Table 2:** Base diet formulation and nutritional levels (%).

Ingredient	Proportion (%)	Calculated nutrient levels	
Corn (CP7.8%)	66.100	CP (%)	17.00
Bran (CP13.6%)	4.100	EE (%)	5.85
Fishmeal (CP68%)	4.500	CF (%)	2.65
Pulped soybean meal (CP46%)	8.200	ASH (%)	5.10
Puffed soya bean (CP36.5%)	9.000	Ca (%)	0.67
Soybean oil	1.500	P (%)	0.56
Fructose	2.000	N-Phy-P (%)	0.36
Glucose	1.000	Lys (%)	1.37
Talcum powder (38%)	0.600	Met (%)	0.50
Calcium hydrogen phosphate	0.800	Cys (%)	0.25
NaCl	0.450	Thr (%)	1.00
L-Lysine hydrochloride	0.670	Trp (%)	0.28
DL-Methionine (99%)	0.210	Arg (%)	1.19
L-Threonine (98.5%)	0.405	Ile (%)	0.60
Tryptophan (98.5%)	0.105	Val (%)	0.75
Choline chloride (50%)	0.150	DE (Macl/kg)	3.48
Phytase 5000	0.020	ME (Macl/kg)	3.20
Antioxidants (30%)	0.020	NE (Macl/kg)	2.45
Mildew inhibitor	0.070		
Multivitamin^1^	0.050		
Complex polymetallic^2^	0.050		
Total	100.00		

1 The premix provides: 4 mg VB1; 8 mg VB2; 6 mg VB12; 3000 IU VD3; 60 IU VE; 4 mg VK3; 0.3 mg Biotin; 50 mg Niacinamide; 30 mg Calcium D-Pantothenate; and 2 mg Folic Acid per kg of ration.

2 The mineral premix provides per kg of full-price feed: 100 mg Fe (FeSO_4_ H_2_O) in the control group; 50 mg Fe (Fe[C2H4O2N]2) in the Fe-Gly group; 5.5 mg Cu ((C2H4NO2)2Cu); 3.5 mg Mn; 90 mg Zn (C4H8N2O4Zn); 0.14 mg I (Ca (IO3)2); 0.275 mg Se (Na2SeO3).

### 2.4 Breeding management

The experiment was conducted at the teaching and research base of the Institute of Animal Nutrition, Sichuan Agricultural University, and the pen was fully disinfected before the start of the experiment. During the test period, we kept the barn ventilated, controlled the temperature of the experimental enclosure at 26–28°C, cleaned the enclosure every day and disinfected it accordingly, fed the piglets three times a day (at 8:00, 14:00 and 20:00), and inspected the piglets every 2 h to observe the feeding situation, and ensured that the residue in the trough of the piglets was not less than 1/4 of the trough’s volume.

### 2.5 Sample collection and processing

#### 2.5.1 Blood sample collection

On the third day after the ETEC infection, blood was collected from the heart of piglets. 1 mL of anticoagulated blood (sodium heparin vacuum blood collection tubes were used for the whole blood analyzer) was collected, and the remaining whole blood was packed in normal vacuum blood collection tubes, centrifuged at 3000 r/min for 15 min to obtain the serum, and the serum samples were stored in separate packages at −20°C until analysis.

#### 2.5.2 Tissue sample collection

After blood collection, all piglets were injected with anesthetics (hypospray), bled to death, and the abdominal cavity of the piglets was quickly dissected to isolate the organs and weigh and measure the lengths of the individual intestinal segments. The duodenum, jejunum, liver, spleen and kidneys were collected, and part of them were preserved in 4% paraformaldehyde solution for subsequent HE, and part of them were frozen in liquid nitrogen and promptly transferred to a −80°C refrigerator for gene and protein analyses; the cecum and colon coeliacs were collected, frozen in liquid nitrogen and promptly transferred to a −80°C refrigerator for preservation.

### 2.6 Growth performance

Piglets were weighed on an empty stomach at 8:00 a.m. on trial days 0, 22 and the day of sampling, and the amount of feed added to each pen each day was recorded, and residual and lost feed in each pen was settled weekly to determine piglet performance at all stages and throughout the whole period of the trial, including the average daily gain (ADG), average daily feed intake (ADFI), and feed gain ratio (F/G).

Average daily weight gain (g/d) = average weight gain (g)/number of feeding days (d).

Average daily feed intake (g/d) = average feed intake (g)/number of feeding days (d).

Feed gain ratio (F/G) = total feed consumption during the test period (g)/piglet weight gain during the test period (g).

### 2.7 Assessment of diarrhea

Piglets were observed and scored for defecation at 8:00, 12:00 and 16:00 every day during the experiment (Table 3). In simple terms: 0 = Normal (Formed or granular), 1 = Mild (soft manure, can be shaped), 2 = Moderately (thick, unformed, no separation of feces and water), 3 = Seriousness (Liquid, unformed, with separation of feces and water) and score ≥ 2 was considered diarrhea.

**Table 3:** Scoring criteria for the degree of diarrhea in piglets.

Fecal appearance	Diarrhea level	Diarrhea score
Formed or granular	Normal	0
Soft manure, can be shaped	Mild	1
Thick, unformed, no separation of feces and water	Moderately	2
Liquid, unformed, with separation of feces and water	Seriousness	3

Diarrhea rate (%) = (days of piglet diarrhea/test days) × 100.

Diarrhea index = sum of diarrhea scores/(number of pigs per pen × number of days of scoring × number of scores per day).

### 2.8 Morphological analysis of the intestinal tract

The jejunal tissue samples fixed in 4% paraformaldehyde were stained with Periodic acid-schif (PAS) after ethanol gradient dehydration, xylene transparency, and immersion wax embedded sections. After sealing the sections, 10 fields of view with intact villi and straight orientation were selected under an Eclipse Ci-L (Nikon, Japan) photomicroscope, and the jejunal villus height and Crypt depth was measured using ImageJ image processing and analysis software.

### 2.9 Determination of non-heme iron in liver, spleen, kidney and each intestinal segment

About 30 mg of tissue was taken and mixed with 1 mL of tissue digestion solution (the formula was a mixture of 3 mol/L hydrochloric acid and 0.61 mol/L trichloroacetic acid, which was dissolved and then fixed to 1 L with deionized water), ensuring that the tip was not contaminated with iron. Subsequently, the tissue was digested at 65°C for not less than 50 h, with shaking 3 times, each time for not less than 10 min, to ensure complete digestion. After digestion was completed, the tissue digest was fixed to 1.5 mL, centrifuged at 10,000 g for 10 min, and 10 μL was pipetted into a 96-well plate, and 200 μL of iron color working solution was added to each well and the color was developed at room temperature for 10 min. Finally, the absorbance value was read using an enzyme marker at 535 nm (13).

### 2.10 Routine blood indicators

Red blood cell (RBC), hemoglobin concentration (HGB), erythrocyte pressure volume (HCV), mean corpuscular volume (MCV), and mean corpuscular hemoglobin content (MCHC) were determined using a fully automated hematology analyzer (Hitachi, Japan).

### 2.11 Serum iron measurement

Serum iron (Serum iron), Unsaturated Iron Binding Capacity (UIBC), Total Iron Binding Capacity (TIBC), and Iron Saturation (TF%) were measured using the Serum Iron Kit (Pointe Scientific™ Iron Standard Reagent) (13).

### 2.12 Statistical analysis

The experimental data were organized and analyzed using Excel for statistical purposes. Statistical analyses were performed using IBM SPSS 27 software, where pre-infection data were analyzed by one-way ANOVA followed by Tukey multiple comparison test; post-infection data were analyzed using two-way ANOVA in a general linear model to assess main effects and interactions. All data are presented as mean ± SEM. Statistical significance was set at *p* < 0.05, and 0.05 < *p* < 0.10 was considered as a trend. Graphs were generated using GraphPad Prism 10.1.2 software.

## 3 Results

### 3.1 The effect of Fe-Gly addition on the growth performance and diarrhea in piglets

Table 4 showed that during 1–21 d of the experiment, piglets in the FeSO_4_ supplementation group exhibited significantly higher ADFI and ADG compared to the control group (*p* < 0.05). As depicted in Table 5, ETEC gavage significantly increased the rate of piglet diarrhea (*p* < 0.05), while the addition of Fe-Gly group effectively alleviated the diarrhea in the infected group, and the addition of Fe-Gly reduced the rate of diarrhea by 12.5% and the diarrhea index by 43.2% compared with the control group.

**Table 4:** Effect of adding Fe-Gly in 1-21d on piglet performance.

Items	Treatment		*p*-values
	100 mg FeSO_4_	50 mg Fe-Gly	
Initial BW Kg	7.05 ± 0.14	7.11 ± 0.13	0.724
21d BW Kg	11.13 ± 0.45	12.64 ± 0.63	0.061
ADFI (Kg/d)	297.8 ± 21.2	389.0 ± 35.3	0.035
ADG (Kg/d)	185.6 ± 17.3	251.2 ± 24.8	0.038
F/G	1.69 ± 0.08	1.59 ± 0.05	0.285
Diarrhea rate (%)	6.82 ± 2.68	4.83 ± 1.63	0.532
Diarrhea index	0.22 ± 0.08	0.17 ± 0.06	0.655

**Table 5:** Effects of added Fe-Gly on piglet performance in 1–24 d.

Items	Treatment				*p*-values		
	100 mg FeSO_4_	50 mg Fe-Gly	100 mg FeSO_4_ + ETEC	50 mg Fe-Gly + ETEC	*P*_1_	*P*_2_	*P_3_*
Initial BW Kg	7.03 ± 0.23	7.24 ± 0.19	7.06 ± 0.16	6.98 ± 0.18	0.729	0.559	0.445
24d BW Kg	11.67 ± 0.68	13.19 ± 0.97	11.67 ± 0.69	13.4 ± 0.97	0.064	0.905	0.898
22d–24d (Post challenge)							
ADFI (g/d)	386.4 ± 36.4	501.6 ± 94.6	390.9 ± 41.9	494.5 ± 55.4	0.086	0.983	0.926
ADG (g/d)	267.5 ± 64.9	301.9 ± 64.1	273.1 ± 43.4	351.3 ± 29.8	0.367	0.658	0.724
Diarrhea rate %	0.00 ± 0.00^b^	0.00 ± 0.00^b^	31.25 ± 13.15^a^	18.75 ± 13.15^ab^	0.481	0.010	0.481
Diarrhea index	0.00 ± 0.00^b^	0.00 ± 0.00^b^	0.88 ± 0.39^a^	0.50 ± 0.34^ab^	0.446	0.010	0.446

*P1 is the P-value comparing the addition of FeSO4 with Fe-Gly, indicating the effect produced by the addition of different iron sources; P2 is the p-value comparing the ETEC infected group with the uninfected group, indicating the effect produced by the infection by ETEC. P3 is the p-value comparing the effect produced by the infection by ETEC in the interaction of the infection by ETEC with the different iron sources. The following is the same.*

### 3.2 The effect of Fe-Gly addition on the organ index of piglets

Table 6 showed that there was no significant difference in the organ indexes of piglets in each group except for the spleen; there was a tendency for an increase in the spleen indexes of piglets in the Fe-Gly added group compared with that of the FeSO_4_ added group (*p* = 0.051); and there was a certain decrease in the spleen indexes of the ETEC infected compared with that of the uninfected group in the Fe-Gly added group (*p* = 0.091).

**Table 6:** Effect of Fe-Gly addition on piglet organ indexes.

Items	Treatment				*p*-values		
	100 mg FeSO_4_	50 mg Fe-Gly	100 mg FeSO_4_ + ETEC	50 mg Fe-Gly + ETEC	*P*_1_	*P*_2_	*P_3_*
Heart index (%)	0.49 ± 0.02	0.49 ± 0.04	0.54 ± 0.02	0.53 ± 0.02	0.714	0.093	0.952
Liver index (%)	2.76 ± 0.11	2.58 ± 0.08	2.51 ± 0.14	2.6 ± 0.14	0.701	0.351	0.269
Spleen index (%)	0.21 ± 0.02^b^	0.28 ± 0.03^a^	0.21 ± 0.01^b^	0.22 ± 0.02^b^	0.051	0.091	0.114
Lung index (%)	1.26 ± 0.06	1.19 ± 0.02	1.24 ± 0.05	1.29 ± 0.03	0.794	0.379	0.154
Kindey index (%)	0.50 ± 0.02	0.55 ± 0.01	0.49 ± 0.03	0.52 ± 0.02	0.098	0.483	0.645

In the row for spleen index, different lowercase letters indicate significant differences between the treatment groups (*P* < 0.05).

### 3.3 The effect of Fe-Gly addition on the morphology of the jejunum in piglets

**Figure 1:**
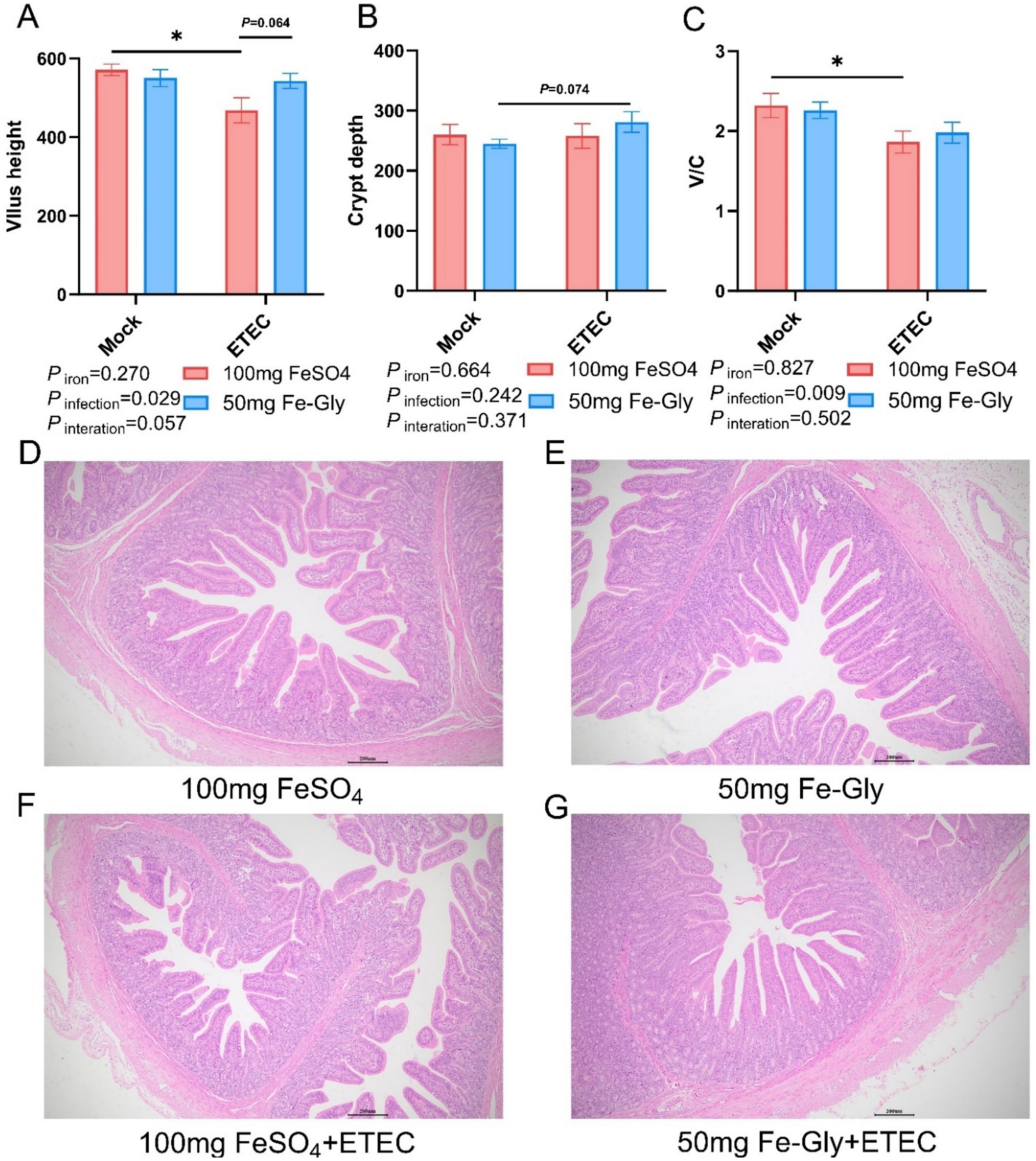
Effect of Fe-Gly addition on the structural morphology of jejunum in piglets. **(A)** Jejunal villus height, *n* = 8; **(B)** Jejunal crypt depth, *n* = 8; **(C)** Jejunal villus to crypt ratio, *n* = 8; **(D–G)** Jejunal pathological sections. Statistical significance is indicated as follows: *P < 0.05.

Figure 1 demonstrated that there was no significant difference in villus height in the Fe-Gly added group compared to the control group (FeSO_4_ added group) in the absence of infection (Figures 1A,D,E). Significant crumpling of the intestinal villi occurred in piglets in the group with 100 mg of added FeSO_4_ after infected (*p* < 0.05), whereas there was no significant change in the intestinal villi in the group with 50 mg of added Fe-Gly (Figures 1A–G). Crypt depth did not change significantly between treatments, but the villus-crypt ratio showed similar differences to villus height (Figures 1B–G). This suggests that the addition of 50 mg of Fe-Gly can alleviate to some extent the damage to the intestinal tract of piglets due to ETEC infection.

### 3.4 The effect of Fe-Gly addition on iron metabolism in the body

**Figure 2:**
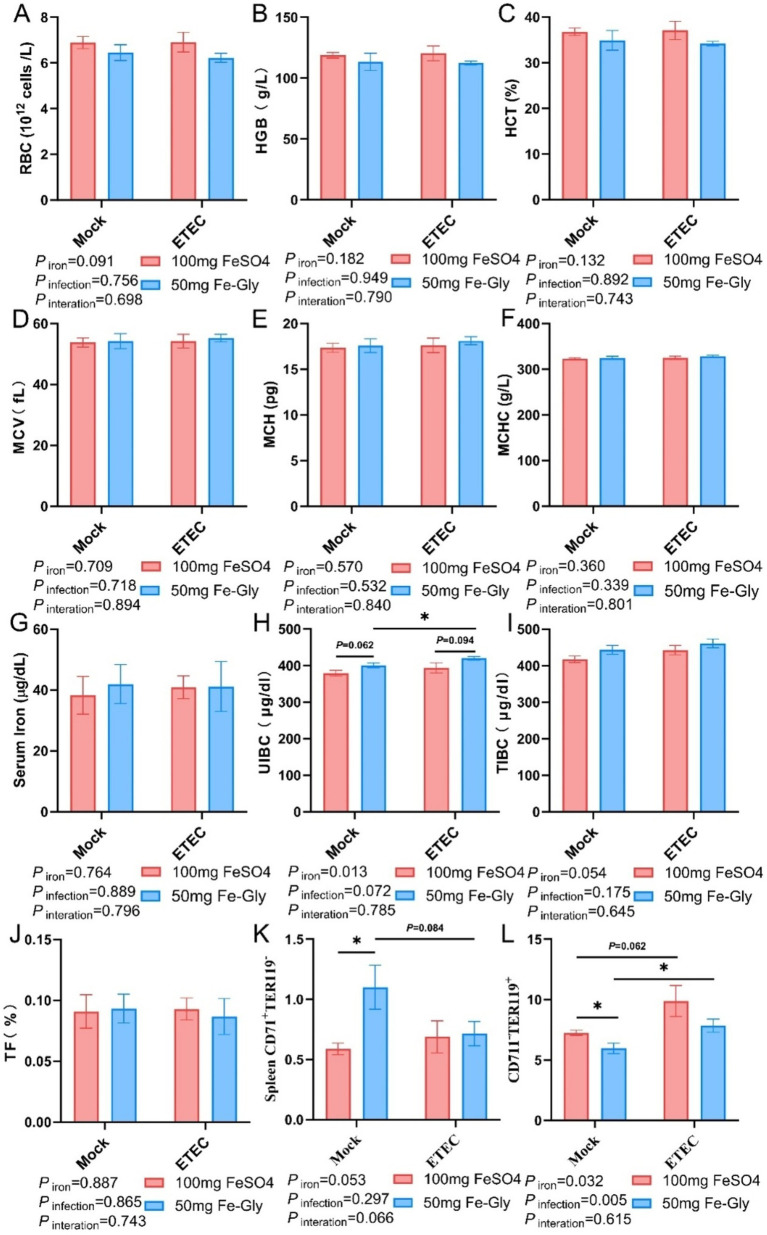
Effect of Fe-Gly addition on hematological and serum iron levels in piglets. **(A)** Erythrocyte number, *n* = 8; **(B)** Hemoglobin, *n* = 8; **(C)** Erythrocyte pressure area, *n* = 8; **(D)** Mean erythrocyte volume, *n* = 8; **(E)** Mean erythrocyte hemoglobin content, *n* = 8; **(F)** Mean erythrocyte hemoglobin concentration, *n* = 8; **(G)** Serum iron, *n* = 8; **(H)** Unsaturated iron-binding capacity, *n* = 8; **(I)** Total iron-binding capacity, *n* = 8; **(J)** Iron saturation, *n* = 8; **(K–L)** Erythroid Progenitor Cells in the spleen, *n* = 8. Statistical significance is indicated as follows: *P < 0.05.

Figure 2 illustrated that in the absence of infection treatment, compared with the control group (FeSO_4_ group), there were no significant differences in blood indices such as RBC, HGB, HCT, MCV, MCH, MCHC, serum iron, UIBC, TIBC, and TF (Figures 2A–J). However, in the Fe-Gly-added group, splenic CD71^+^TER119^−^ cells were significantly increased, and CD71^−^TER119^+^ cells were significantly reduced (*p* < 0.05, Figures 2K,L). After the infection, UIBC was significantly higher in the group with 50 mg of Fe-Gly (*p* < 0.05), whereas there was no significant change in the group with 100 mg of FeSO_4_ (Figure 2H). In the Fe-Gly group, there was a significant increase in splenic CD71^−^TER119^+^ cells (*p* < 0.05, Figure 2H).

### 3.5 Effect of adding Fe-Gly on the iron content of various tissues in piglets

**Figure 3:**
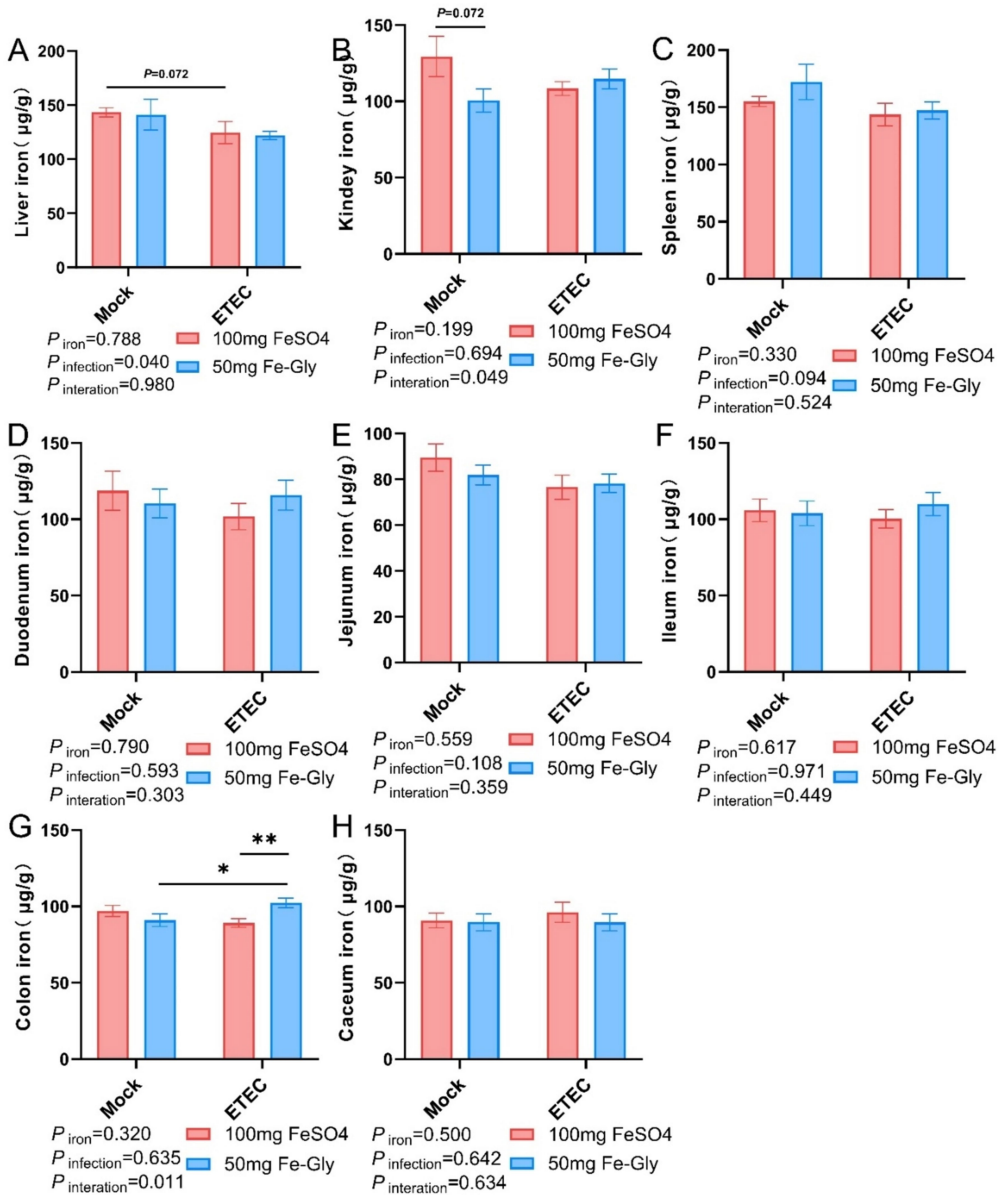
Effect of Fe-Gly addition on iron content of piglets in various tissues. **(A)** Hepatic non-heme iron levels, *n* = 8; **(B)** Renal non-heme iron levels, *n* = 8; **(C)** Splenic non-heme iron levels, *n* = 8; **(D)** Duodenal non-heme iron levels, *n* = 8; **(E)** Jejunal non-heme iron levels, *n* = 8; **(F)** Ileal non-heme iron levels, *n* = 8; **(G)** Colonic non-heme iron levels, *n* = 8; and **(H)** Cecum non-heme iron levels, *n* = 8. Statistical significance is indicated as follows: *P < 0.05, **P < 0.01.

Figure 3 revealed that the addition of Fe-Gly decreased the iron content in the liver and kidney compared with the control group, while there was no significant effect on the iron content in other tissues. In the toxicity group, the addition of Fe-Gly can increase the iron content in the duodenum, jejunum, ileum and colon to a certain extent, indicating that the addition of Fe-Gly in the ration can alleviate the damage caused by ETEC, and still effective intake of iron sources to meet the body’s needs.

### 3.6 Expression of genes related to duodenal iron metabolism

**Figure 4:**
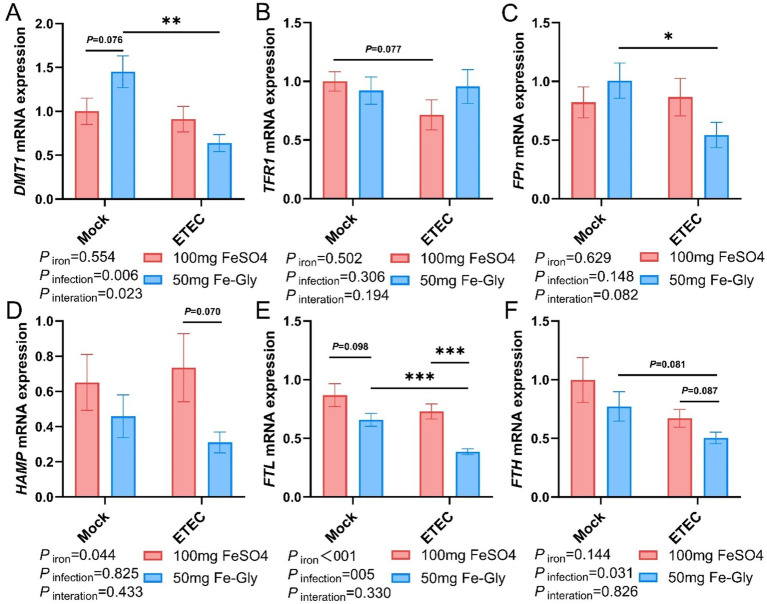
Effect of Fe-Gly addition on the expression of genes related to iron metabolism in the duodenum of piglets. **(A)** Divalent metal transporter 1 (*DMT1*), *n* = 8; **(B)** Transferrin receptor 1 (*TFR1*), *n* = 8; **(C)** Ferroportin (*Fpn*), *n* = 8; **(D)** Hepcidin antimicrobial peptide (*HAMP*), *n* = 8; **(E)** Ferritin light chain (*FTL*), *n* = 8; **(F)** Ferritin heavy chain (*FTH*), *n* = 8. Statistical significance is indicated as follows: *P < 0.05, **P < 0.01, ***P < 0.001.

**Figure 5:**
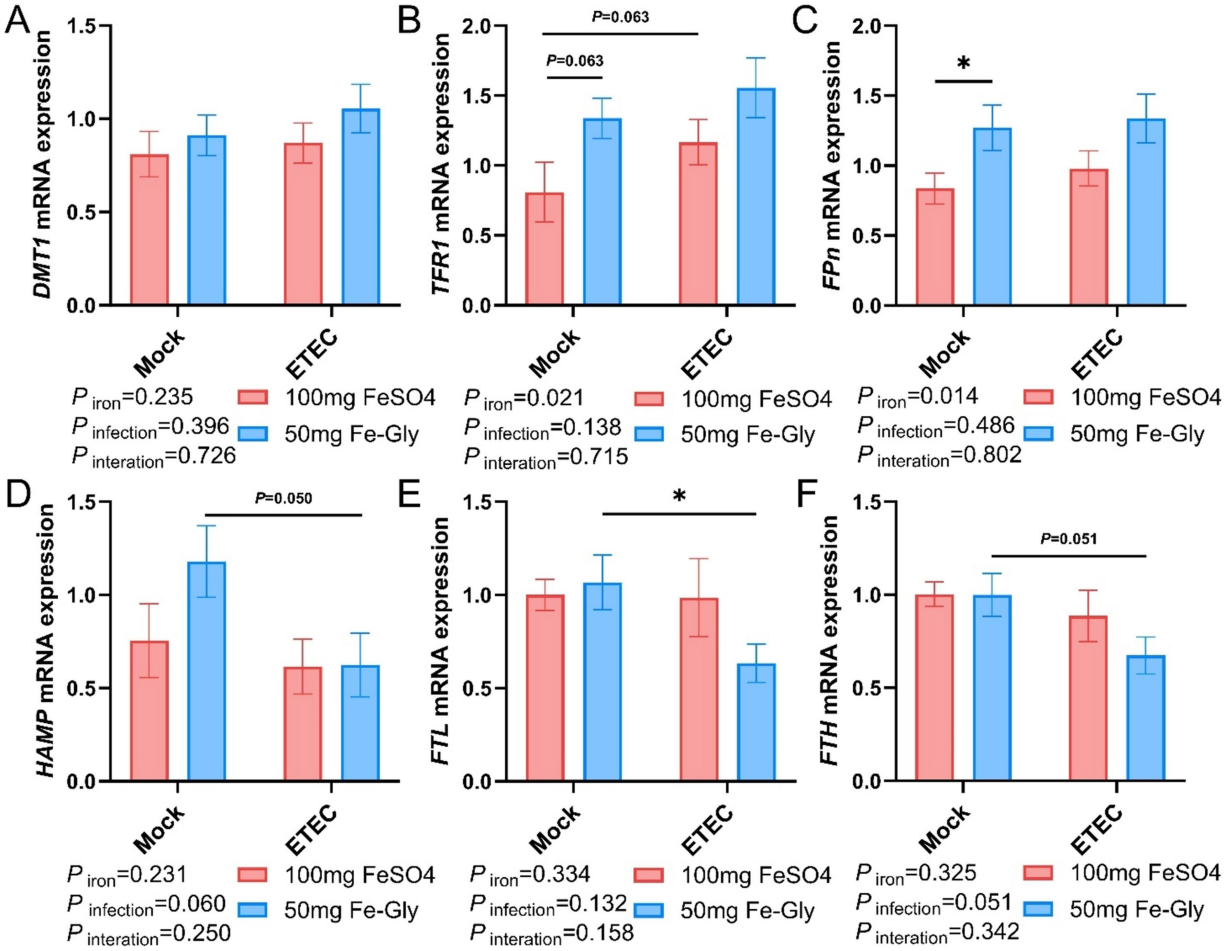
Effect of Fe-Gly addition on the expression of iron metabolism-related genes in piglets’ liver. **(A)** Divalent metal transporter 1 (*DMT1*), *n* = 8; **(B)** Transferrin receptor 1 (*TFR1*), *n* = 8; **(C)** Ferroportin (*Fpn*), *n* = 8; **(D)** Hepcidin antimicrobial peptide (*HAMP*), *n* = 8; **(E)** Ferritin light chain (*FTL*), *n* = 8; **(F)** Ferritin heavy chain (*FTH*), *n* = 8. Statistical significance is indicated as follows: *P < 0.05.

Figure 4 demonstrated that in the absence of infection treatment, compared with the control group (the group with added FeSO_4_), in the group with added Fe-Gly, the expression of duodenal *DMT1* gene tended to be elevated (*p* = 0.08, Figure 4A), while the rest of the genes did not show significant differences (Figures 4A–F). After the infected treatment, the expression of duodenal *TFR1* gene tended to decrease by the addition of FeSO_4_ group (*p* = 0.08, Figure 4B); the addition of Fe-Gly group showed a significant decrease of duodenal *DMT1* gene (*p* < 0.01, Figure 4A), the expression of *FPN* gene was significantly decreased (*p* < 0.05, Figure 4C), and the expression of *FTL and FTH* gene expression tended to decrease (Figures 5E,F). Under infected treatment, compared with the control group, the addition of Fe-Gly group showed a highly significant decrease in the expression of *FTL* of duodenal genes (*p* < 0.001, Figure 4E), and there was a tendency for a decrease in the expression of *FPN*, *HAMP*, and *FTH* genes, but there was no significant difference (Figures 4C,D,F).

### 3.7 Expression of genes related to hepatic iron metabolism

Figure 5 indicated that in the case of no infected treatment, compared with the control group (the group with added FeSO_4_), in the group with added Fe-Gly, the expression of hepatic *FPN* gene was significantly elevated (Figure 5C), and the expression of *TFR1* gene tended to be elevated (*p* = 0.063, Figure 5B). After the infection, in the Fe-Gly addition group, the expression of hepatic *FTL* gene was significantly decreased (*p* < 0.05, Figure 5E), the expression of HAMP gene tended to decrease (*p* = 0.050, Figure 5D), and the expression of *FTH* gene had a tendency to decrease (*p* = 0.051, Figure 5F). In the FeSO_4_ addition group, the expression of hepatic genes did not show any significant change in all groups (Figures 5A–F). These results suggest that the addition of Fe-Gly can partially modify the effect of ETEC infection on hepatic iron metabolism.

### 3.8 Expression of genes related to jejunal iron metabolism

**Figure 6:**
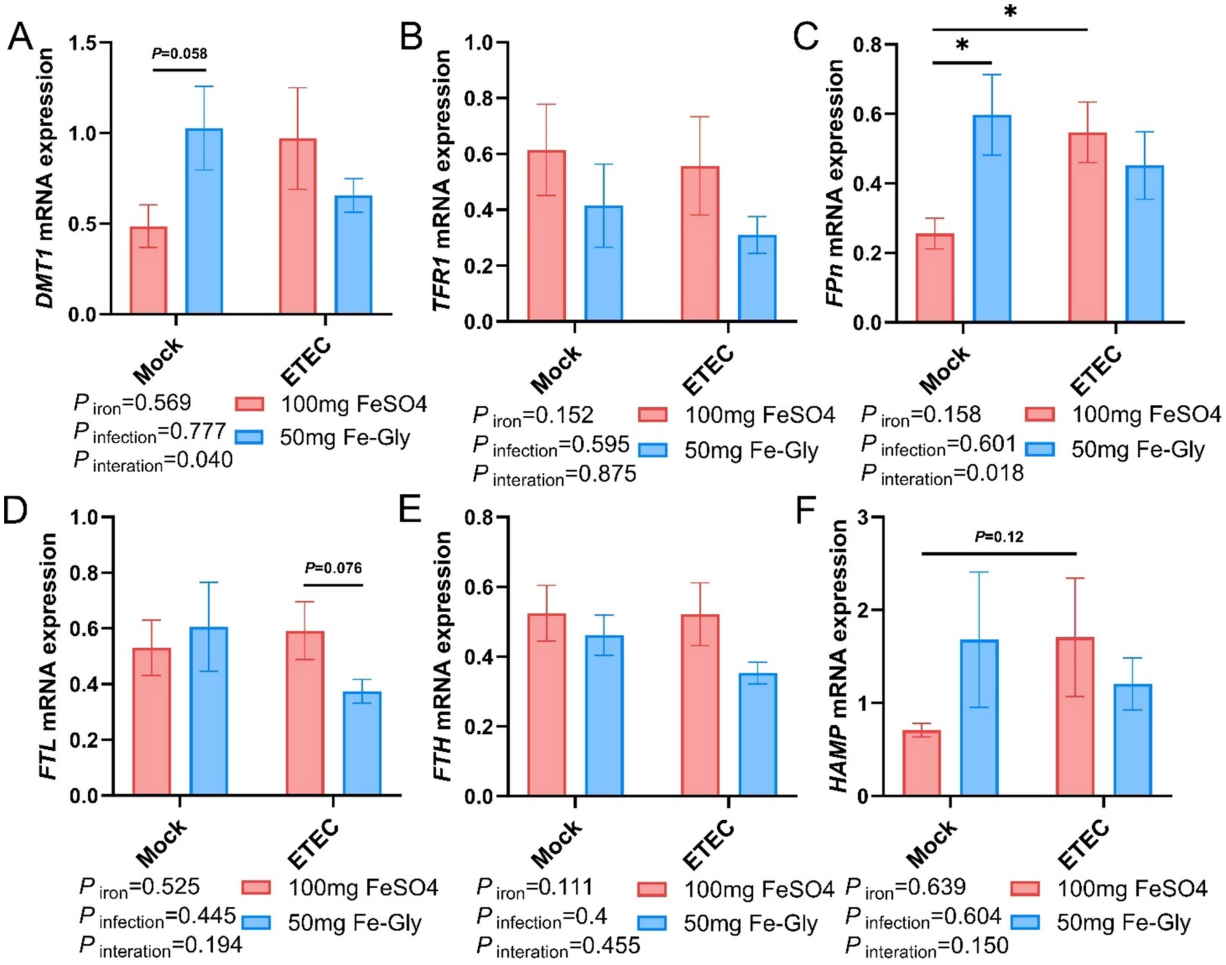
Effect of Fe-Gly addition on the expression of genes related to iron metabolism in the jejunum of piglets. **(A)** Divalent metal transporter 1 (*DMT1*), *n* = 8; **(B)** Transferrin receptor 1 (*TFR1*), *n* = 8; **(C)** Ferroportin (*Fpn*), *n* = 8; *n* = 8; **(D)** Ferritin light chain (*FTL*), *n* = 8; **(E)** Ferritin heavy chain (*FTH*), *n* = 8, **(F)** Hepcidin antimicrobial peptide (*HAMP*). Statistical significance is indicated as follows: *P < 0.05.

Figure 6 revealed that the expression of jejunal *FPN* gene was significantly higher in the Fe-Gly group compared with the control group (FeSO_4_ group) in the absence of infection (*p* < 0.05, Figure 6C). The addition of Fe-Gly group enhanced the expression of the jejunal *DMT1* and *HAMP* genes in the absence of infection compared with the control (FeSO_4_ group) (Figures 6A,F); the expression of jejunal *FPN* gene was significantly lower in the 100 mg of FeSO_4_ group after infected (*p* < 0.05, Figure 6C); the expression of jejunal *FPN* gene was significantly lower in the 100 mg of FeSO_4_ group compared with the control (FeSO_4_ group) in the presence of infected. After the addition of 100 mg of FeSO_4_, the expression of jejunal *FPN* gene was significantly reduced (*p* < 0.05, Figure 6C). Under infection conditions, the addition of Fe-Gly group showed a tendency to reduce the expression of jejunal *FTL* gene compared with the control group (*p* = 0.076, Figure 6D). For the expression of jejunal *TFR1* and *FTH* genes, there were no significant differences among treatments (Figures 6B,E).

### 3.9 Expression of jejunal inflammatory factor-related genes

**Figure 7:**
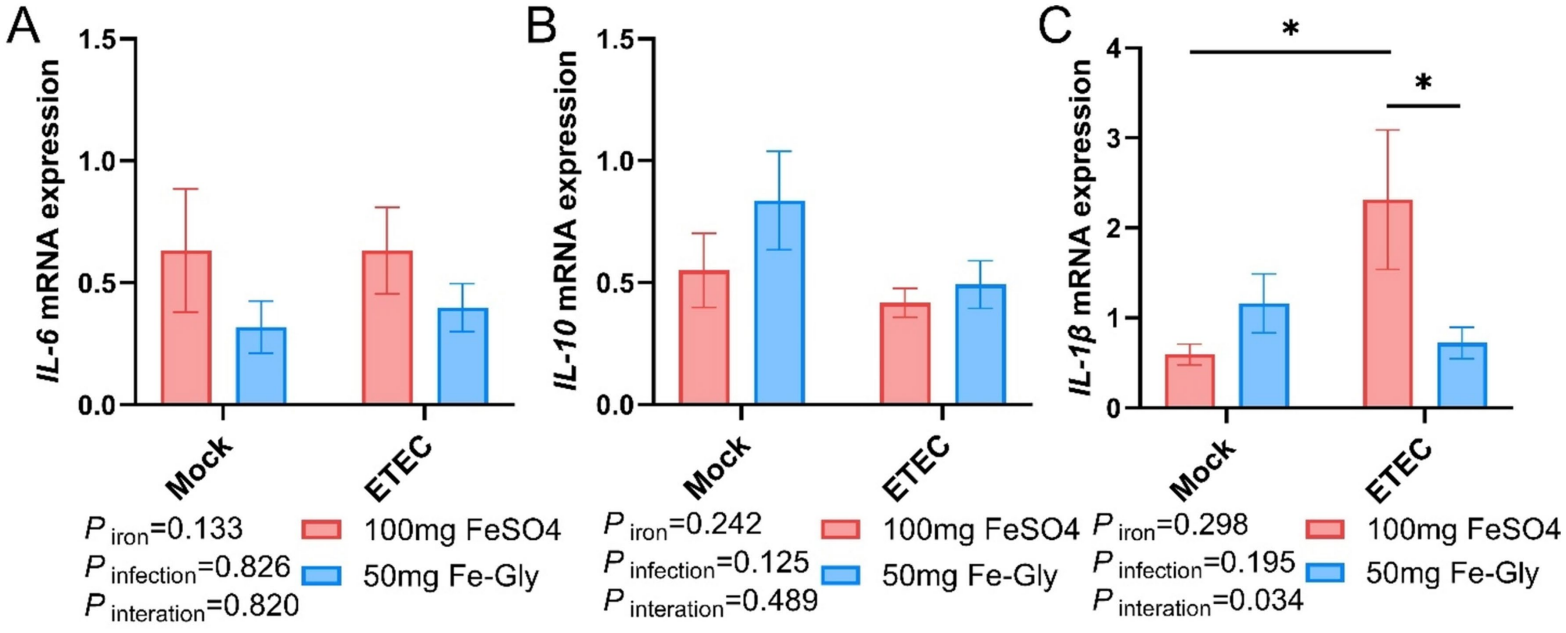
Effect of Fe-Gly addition on jejunal inflammation gene expression in piglets. **(A)** Interleukin 6 (*IL-6*), *n* = 8; **(B)** Interleukin 10 (*IL-10*), *n* = 8; **(C)** Interleukin 1β (*IL-1β*), *n* = 8. Statistical significance is indicated as follows: *P < 0.05.

Figure 7 demonstrated that regarding the expression of jejunal *IL-6* gene, there was no significant difference between the treatment groups, but in both control and infected groups, the addition of Fe-Gly decreased the expression of jejunal *IL-6* gene compared to the addition of FeSO_4_ (Figure 7A). After infection, the addition of FeSO_4_ significantly increased in the expression of jejunal *IL-1β* gene (*p* < 0.05, Figure 7C); in the infected treated group, the addition of Fe-Gly significantly increased in the expression of jejunal *IL-1β* gene compared with the control group (*p* < 0.05, Figure 7C). These results suggest that the addition of 50 mg of Fe-Gly could alleviate the inflammatory response induced by ETEC infection on the organism to a certain extent.

## 4 Discussion

The weaning stage of piglets is often accompanied by changes in the environment and dietary structure, leading to a series of stress reactions (14). Sow milk has low iron content, and piglets can obtain less iron from their mother. Additionally, the liver iron storage of newborn piglets is low, which cannot meet the demand for rapid growth during the early stage (15). Therefore, exogenous iron supplementation is usually performed for newborn piglets, while adding easily absorbed iron to the feed is the primary method to prevent iron deficiency in weaned piglets (16, 17). Li Jiangtao et al. found that the effect of adding 50 mg/kg of Fe-Gly to the diet was better than that of adding 150 mg/kg of FeSO_4_ in terms of AGD, ADFI, and F/G, with a decreasing trend observed as the amount of Fe-Gly increased (18). Feng et al. reported that the addition of Fe-Gly at a level of 90 mg/kg in diets was superior than the addition of 120 mg/Kg of FeSO_4_ (9). Ma et al. demonstrated that Fe-Gly in the diet significantly increased ADFI and ADG while significantly reducing early diarrhea in piglets (19). Sun Limei et al. found that the supplementation of small peptide chelated iron in the ration could increase iron storage in animals and improve intestinal barrier function and immune function (20). We obtained similar results regardless of whether ETEC infection was present or absent. In this study, the addition of 50 mg of Fe-Gly significantly increased ADFI and ADG, showing no difference compared to the 100 mg/kg FeSO_4_ addition group (Table 4). The inclusion of Fe-Gly in the ration improved piglet growth performance and reduced the incidence of diarrhea, which is completely consistent with previous research. Furthermore, the addition of 100 mg of FeSO_4_ and 50 mg of Fe-Gly to the diets had no significant effect on iron deposition in the tissues (Figure 3). These results further demonstrate that adding Fe-Gly to the diet can effectively enhance the growth performance of weaned piglets. In addition, the high bioavailability of Fe-Gly can significantly reduce iron excretion, thereby decreasing environmental pollution.

Iron in food is primarily absorbed into intestinal cells in the duodenum via DMT1, and then pumped into the bloodstream by *FPn*, where it is transported to the liver for storage (21). Our research found that the addition of 50 mg of Fe-Gly to the feed significantly increased the gene expression of *DMT1* in the duodenum under non-infected conditions (Figure 4). This result indicates that Fe-Gly is more easily absorbed in the body compared to FeSO_4_, and the increase in serum iron and TIBC further supports this notion (Figure 2). This is consistent with previous reports that the absorption efficiency of Fe-Gly in the intestine is significantly higher than that of inorganic iron (19). This explains why the addition of 50 mg/kg of Fe-Gly can achieve effects equivalent to 100 mg/kg of FeSO_4_, and even better results. Typically, unabsorbed iron in the duodenum enters the hindgut along with the chyme (22). Since microorganisms have a much greater capacity to utilize iron than the host itself, the accumulation of iron in the hindgut will significantly reduce the abundance of harmful bacteria while promoting the growth of pathogenic bacteria, thereby increasing intestinal inflammation (23, 24). For example, in ETEC, siderophores are transported across the double membrane envelope via a gating mechanism that links the inner and outer membranes. This mechanism ensures that siderophores can effectively capture iron from the external environment and transport it into the cell to meet the physiological needs of the cell. In this way, bacteria can survive and reproduce under conditions of iron scarcity, while also demonstrating the complex adaptive mechanisms of microorganisms in iron acquisition (23). Additionally, this can also enhance the infection of pathogenic microorganisms, leading to serious diarrhea in piglets (25). The results of this study found that, the addition of 50 mg of Fe-Gly was effective in decreasing the levels of iron in the cecum and colonic. Notably, compared to the control group, ETEC infection significantly increased the incidence of diarrhea in piglets and proinflammatory cytokine *IL-1β* gene expression of jejunum (Figure 7). In contrast to the FeSO_4_ addition group, the feed supplemented with Fe-Gly effectively reduced the incidence of diarrhea in piglets and *IL-1β* gene expression of jejunum. This demonstrates that adding a lower dose of Fe-Gly to the feed can effectively alleviate diarrhea in piglets and intestinal inflammation caused by ETEC infection without affecting their growth performance. This may be related to the reduced dosage of Fe-Gly, the lower iron content in the intestine, and the high absorption efficiency of Fe-Gly.

The intestinal tract is the most important digestive organ in the organism, playing a crucial role in maintaining the homeostasis of the organism (26). Among them, the morphology and structure of the small intestine, as well as its functional integrity, play an important role in the maintain the health of the animal organism (27). Intestinal villi in the small intestine’s wrinkled wall greatly increase the area for nutrient absorption, and their height and health determine the efficiency of digestion and absorption in animals (28). Increased villus height indicates stronger absorption function, while decreased height signifies fewer absorption-related cells, weakening this function (29). Previous studies have shown that ETEC infection in weaned piglets can lead to crumpling of small intestinal villi, a decrease in villus height, a significant increase in crypt depth, and changes in intestinal morphology, which in turn affect the body’s nutrient absorption efficiency (30, 31). Studies have found that Fe-Gly can improve gut barrier function by regulating the composition of the gut microbiome, increasing the relative abundance of beneficial bacteria, and decreasing the relative abundance of potentially pathogenic bacteria (32). After ETEC infection, piglets in the FeSO_4_ group showed a significant decrease in villus height and no significant change in crypt depth, resulting in a reduced villus-crypt ratio. In contrast, there were no significant changes in villus height and crypt depth in the Fe-Gly group. This may be due to the low dosage of Fe-Gly added, which does not produce ionic iron during the absorption process. Consequently, absorption efficiency was significantly improved, reducing the iron available to the intestinal microorganisms, inhibiting the proliferation of ETEC, and thereby mitigating intestinal damage and promoting intestinal integrity of the piglets.

## 5 Conclusion

Adding 50 mg Fe-Gly to the diet can significantly increase ADFI and ADG, thereby promoting piglet performance to a certain extent and alleviating issues related to piglet diarrhea and reduced performance caused by ETEC infection. Furthermore, the addition of Fe-Gly can also increase the level of TIBC in the serum of the piglets, enhancing their ability to bind and transport iron. Fe-Gly can also mitigate the damage to the jejunal segment caused by ETEC infection to some extent. In summary, adding 50 mg of Fe-Gly not only meets the daily iron requirements of piglets but also improves the efficiency of iron utilization. This addition reduces residual pathogenic ETEC iron in the intestine, thereby decreasing the availability of iron to pathogenic microorganisms, inhibiting the proliferation of intestinal pathogens, and protecting the intestinal health of piglets. Additionally, it can significantly reduce environmental pollution. By minimizing the sources of unutilized iron, the use of Fe-Gly helps to lessen the negative impact on the environment and promotes sustainable farming practices.

## Data Availability

The original contributions presented in the study are included in the article/supplementary material, further inquiries can be directed to the corresponding authors.
